# Pakistan’s Performance in Global Impact Factor Race

**DOI:** 10.12669/pjms.344.16035

**Published:** 2018

**Authors:** Sultan Ayoub Meo, Shaukat Ali Jawaid

**Affiliations:** 1 Sultan Ayoub Meo, MBBS, M Med Ed, Ph.D, FRCP (London), FRCP (Dublin), FRCP (Glasgow), FRCP (Edinburgh) Profesor and Consultant, Department of Physiology and Medical Education, College of Medicine, King Saud University, Riyadh, Saudi Arabia. E-mail: sultanmeo@hotmail.com smeo@ksu.edu.sa; 2 Shaukat Ali Jawaid Chief Editor, Pakistan Journal of Medical Sciences, Karachi - Pakistan, Secretary, Eastern Mediterranean Associaton of Medical Editors (EMAME) E-mail: pulse@pulsepakistan.com pjms@pjms.com.pk

Research in science and social sciences play a vital role in education, planning, decisions, social and economic progress along with long-standing sustainable developments.^1^ The innovative research in science and technology contribute to improve the living standards and an excellence of life. Scientific publications reveal the academic and scientific advancement and science journals have significant impact in exchange of knowledge both at national and international levels.^2^-^3^

Pakistan is a home to more than 200 million people, 189 Higher Education Commission (HEC) chartered universities and degree awarding institutes^4^ including 29 medical universities, 157 medical schools^5^ 125 engineering, 92 management sciences and 28 agricultural institutes.^4^

In Pakistan, there are 371 HEC indexed journals in various academic disciplines of science and social sciences^4^. In the last week of June 2018, Philadelphia USA based, a notable indexing institute, Thomson Reuters, Institute of Scientific Information (ISI) Web of Science, currently known as “Clarivate Analytics” released a global science and social science journals Impact Factor (IF) list of year 2017.^6^

Impact factor represents the total number of citations to a journal’s articles divided by the number of articles published during the previous two years. It is widely used in the academic world as a yardstick of a journal’s prestige. From Pakistan, out of 371 only 12 (3.24%) academic journals have achieved a place in ISI-Web of Science.

Worldwide, 12271 science and social sciences journals are indexed in the ISI-Web of Science, their IF is ranging from 0.001 to 244.58.^6^ The Cancer Journal for Clinicians USA achieved a top position in the world with Impact Factor 244.58. The other top ranking journals are New England Journal of Medicine USA 79.25; Lancet USA 53.24; Nature UK 41.57; and Science USA 41.05.^6^ These journals are leading the world and have maintained their topmost positions in the global IF race.

In our environs, China is leading the region with 203 academic journals achieved a remarkable position in ISI Web of science with IF 0.0045 to 15.393. India has 104 with IF 0.096 to 2.658; Iran 42 IF 0.280 to 2.667; Pakistan 12 IF 0.280 to 1.217; and Bangladesh has 4 with IF 0.214 to 1.532. Only one Journal from Pakistan, “Pakistani Veterinary Journal” exceeds the IF 1.217.^6^

While comparing the quartile factor of the journals, subject category in percentile rank, the top 25% of journals in a particular category are placed in Q1, next in Q2 and so on. 41 Chinese journals achieved a position in first quartile Q1, Q2: 63, Q3:57 and 62 journals in Q4. India has Q1: 0, Q2: 4, Q3: 26 and in Q4:74. Iran has 42 academic journals from them Q1: 1, Q2: 4, Q3: 12 and in Q4:25. However, Bangladesh has 4 ISI-Web of Science indexed journals only one journal placed a position in Q3 and 3 in Q4.^6^

The quartile ranking of Pakistani journals is: 2 journals in both Q2 and Q3 and the remaining 8 journals are in Q4. Only one Journal, Pakistan Veterinary Journal exceeds the IF 1.217 and two journals placed a position in quartile 2.^6^

In medical sciences, Pakistan Journal of Medical Sciences achieved an IF 0.719; Journal of Pakistan Medical Association IF 0.718; Journal of College of Physicians and Surgeons of Pakistan- JCPSP IF 0.439.^6^ These medical sciences journals are establishing a platform to publish quality research but still the road is rutted and needs its renewal. They should fascinate the international science community and enhance the research visibility in the global science to upsurge the IF and quartile ranking of the academic journals to compete internationally. Sadly, few Pakistani journals, which are celebrating their golden anniversaries of 50 years, have yet still failed to achieve a place in ISI Web of Science.

Editing and publishing a quality peer reviewed journal is a full time job and a team work but a vast majority of medical journals published form Pakistan have part time editors. Most medical universities, medical institutions and professional specialty organizations publishing journals have given this additional responsibility to busy clinicians and medical teaching faculty members who are not provided needed facilities. Hence, they cannot be expected to devote enough time. This is one of the reasons for the present dismal state of affairs and why more journals from Pakistan have so far failed to get indexed and earn an Impact Factor through coverage by Web of Sciences.^7^,^8^

Despite lot of criticism Impact Factor and Indexation in Medline are considered as an indication of quality of manuscripts and standard of a Journal. However, it is not and should not be the only criteria to judge the standard of a journal.^9^ In the past questions have also been raised frequently about the way IF is calculated and there was an interesting debate on WAME and Declaration on Research Assessment (DORA), USA about use of Impact Factor.^10^ It is also alleged that IF is threatening to skew the course of scientific research. Some researchers now have inclination to select topics for research which will be readily accepted by the high Impact Factor Journals rather than topics which fulfil the practical needs of the society and a particular community. Some Editors have already started to manipulate their Journal Impact Factor through deceptive practices to inflate their own ranking by asking the authors to cite papers published in their journal which is clear violation of scientific ethics.^11^

There are several strategies which, if employed may increase the Impact Factor of a journal. Despite misuse and abuse, the Journal Impact Factor will retain its impact and won’t fade away soon. Journal editors and Publishes anxiously await the publication of Journal Citaiton Report released every year by Thompson Reuters Web of Sciences now renamed as Clarivate Analytics to know their Impact Factor.^11^

As stated earlier, unfortunately there are only three medical journals in Pakistan which enjoy Impact Factor^6^ and they are under tremendous pressure from the authors to publish their manuscripts since the regulatory agencies particularly Higher Education Commission has a requirement to publish manuscripts in Impact Factor Journals. HEC should help more and more journals get an Impact Factor. However, the policies being pursued by the HEC at present of giving financial grants to various journals is not improving the situation. It has failed in the past and is bound to fail in future as well. It will be much better if instead of giving grants, HEC and other regulatory agencies in Pakistan could provide facilities to the Journals for training the Editors, support staff by competent professionals not the way it has been doing in the past, help them design the journal websites, to acquire and use some manuscript management system like Open Journal System or Editorial Manager etc. They can also facilitate the journals by providing the software or service to generate XML files for submission to PubMed Central which will go a long way in improving the visibility of Pakistani medical journals. We can learn some lesson from our neighbouring countries; India and Iran. India has 104 and Iran has 42 journals^6^ with Impact Factor simply because; they have some well trained distinguished Editors as its members and they have been helping, guiding them besides providing facilities for training as well as Indexing which made all the difference. To the best of our knowledge HEC does not have any professional medical editor as a Member of the Journal Evaluation Committee, hence how they can be expected to guide and assist the medical journals. The experts, HEC had selected in the past to run training courses were just an apology about which less said the better. It was highly disappointing for a few distinguished editors who were attending the course and this was also pointed out to the authorities concerned.^12^,^13^

**Figure 1:**
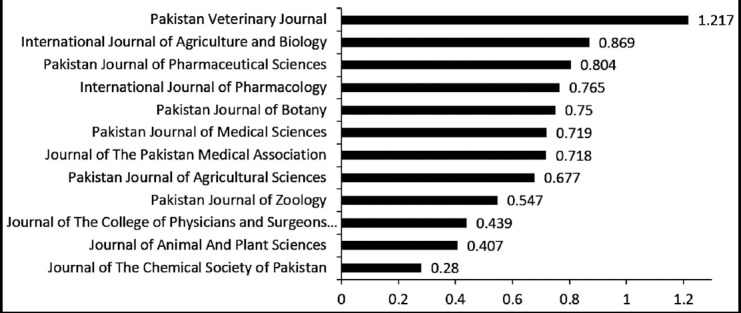
Impact Factor of Pakistani Journals indexed in ISI-Web of Science 2018.

**Figure 2:**
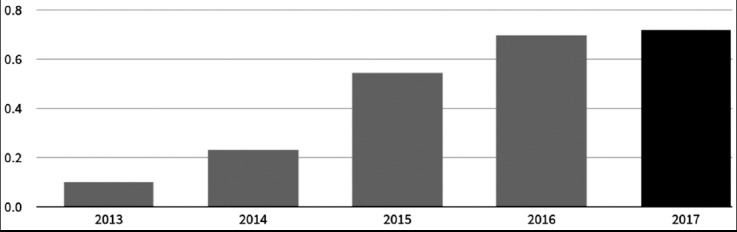
Increasing Impact Fator of Pakistan Journal of Medical Science from 2013 to 2017

There are many science metrics, including Impact Factor, C-index, h-index, Matthew value, Eigen factor, Article influence,^2^ but each of them has its own strength and limitation. IF has an impact in scientific community while taking decisions about where to publish, to promote, whom to hire and fire and to receive the research grants.^2^ There is a great criticism and debate about the dominancy of IF to measure the science, but still the large part of the science community believes that IF is a powerful tool for the global evaluation of the scientific quality and visibility. Hirsch Index (h-Index) measures the productivity and citation impact of an individual and IF is a measure of a journal quality and productivity. Hirsch Index is gaining popularity in science community, although this scientific measuring tool is passing in its adolescent age, but with the passage of time, it will be a more powerful metric to measure the scientific credentials and science among the scientists.

Undoubtedly, Pakistan has great talent, but the academic and research institutes leaderships’ priorities to grasp the importance of indexing of academic journals are extremely lacking. HEC has to establish policies for the hiring and promotions of the faculty members as well as the head of the academic institutes to publish in ISI Web of science indexed journals to enhance the visibility of science. It is time for change, to understand the worth of scientific research and its impact on economy, development and political stability. Pakistan should promote collaboration with the talent-rich international institutes and share novel research in leading science journals to enhance the scientific visibility of the institutes as well as the nation. Moreover, we need to strengthen the regulatory bodies like HEC and PM&DC who should acquire the services of both national and international professional medical editors to guide and train to frame the terms of reference for evaluation of biomedical journals to help more Pakistani medical journals acquire an Impact Factor. Pakistan must attract the best, bright brains back to the placental place to boost education, research, science, and technology in the region and shift the state towards knowledge based economy.
